# Reactive Oxygen Species-Sensitive Nanophotosensitizers of Methoxy Poly(ethylene glycol)-Chlorin e6/Phenyl Boronic Acid Pinacol Ester Conjugates Having Diselenide Linkages for Photodynamic Therapy of Cervical Cancer Cells

**DOI:** 10.3390/ma15010138

**Published:** 2021-12-25

**Authors:** Ju-Il Yang, Hye-Lim Lee, Seon-Hee Choi, Jungsoo Kim, Young-Bob Yu, Young-IL Jeong, Dae-Hwan Kang

**Affiliations:** 1 Department of Medical Science, Pusan National University School of Medicine, Yangsan 50612, Korea; yangjuil@outlook.kr; 2 Department of Internal Medicine, Pusan National University Yangsan Hospital, Yangsan 50612, Korea; 3 Research Institute of Convergence of Biomedical Sciences, Pusan National University Yangsan Hospital, Yangsan 50612, Korea; roasua@hanmail.net (H.-L.L.); jsmsmo@naver.com (S.-H.C.); jskimpnuh@naver.com (J.K.); 4 Department of Emergency Medical Rescue, Nambu University, Gwangju 62271, Korea; ybyu@nambu.ac.kr; 5 Department of Herbal Pharmaceutical Development, Nambu University, Gwangju 62271, Korea

**Keywords:** nanophotosensitizer, ROS-sensitive, photodynamic therapy, cervical cancer, chlorin e6

## Abstract

The aim of this study is to fabricate nanophotosensitizers composed of methoxy poly(ethylene glycol) (mPEG), chlorin e6 (Ce6), and phenylboronic acid pinacol ester (PBAP) with diselenide linkages for reactive oxygen species (ROS)-sensitive photodynamic therapy (PDT) of cervical cancer cells. To fabricate nanophotosensitizers, Ce6 was conjugated with mPEG via selenocystamine linkage and then remaining carboxylic acid groups of Ce6 was attached to PBAP (mPEGseseCe6PBAP conjugates). Nanophotosensitizers of mPEGseseCe6PBAP conjugates were prepared by dialysis method. In transmission electron microscope (TEM) observation, nanophotosensitizers of mPEGseseCe6PBAP conjugates have spherical shapes and their diameters were less than 150 nm. The average diameter of mPEGseseCe6PBAP nanophotosensitizers was 92.7 ± 9.6 nm in particle size analysis. When H_2_O_2_ was added to the nanophotosensitizer solution, nanophotosensitizers were sensitively disintegrated according to the H_2_O_2_ concentration and then changed from monomodal distribution to multimodal distribution in particle size distribution. Furthermore, Ce6 release from nanophotosensitizers also increased according to the H_2_O_2_ concentration. When H_2_O_2_ was added to cell culture of HeLa human cervical cancer cells, intracellular Ce6 uptake of nanophotosensitizers were gradually increased according to the H_2_O_2_ concentration, indicating that nanophotosensitizers showed ROS-sensitive delivery of Ce6 against cancer cells.As well as free Ce6, nanophotosensitizers in the absence of light irradiation have low intrinsic cytotoxicity against RAW264.7 cells and HeLa cells. However, nanophotosensitizers induced cell death dose-dependently under light irradiation. Especially, nanophotosensitizers showed significantly higher ROS generation and phototoxicity against HeLa cells in vitro. When nanophotosensitizers were intravenously administered to animal tumor xenograft model of HeLa cells, tumor tissues revealed stronger fluorescence intensity than other tissues by light irradiation while absence of light irradiation induced relatively lower fluorescence intensity in tumor tissues, indicating that nanophotosensitizers have sensitivity against oxidative stress in tumor tissues. We suggest that nanophotosensitizers of mPEGseseCe6PBAP conjugates are promising vehicle for PDT of cervical cancer cells.

## 1. Introduction

PDT using photosensitizers is believed to be a safe option for treatment of malignant disorders [1,2,3]. Since photosensitizers are only activated in the light irradiation condition and then generated reactive oxygen species (ROS), they are nontoxic to normal cells or tissues in the absence of light irradiation [3,4,5]. Especially, PDT has advantages for treatment of squamous or epithelial phenotype of tumors since the penetration depth of light is limited below 15 mm and the photosensitizer efficiently produces ROS within the limits of light irradiation depth [5,6,7]. For example, PDT is considered as an effective treatment option for skin cancer such as superficial basal cell carcinoma since local drug delivery is believed to be a promising candidate for skin cancer instead of a systemic approach [8,9,10]. Kim et al., reported that 5-amino levulinic acid (5-ALA)-mediated PDT generated oxidative stress in cholangiocarcinoma cells by production of ROS and then efficiently inhibited the growth of tumors [11]. The PDT approach is also promising treatment option for squamous cervical cancer phenotype since the most phenotype of cervical cancer is known to squamous cell carcinoma [12]. In spite of these advantages, the current approach of PDT using traditional photosensitizers still has drawbacks for application in clinical stage. For example, penetration and/or transport efficacy of water-soluble photosensitizers such as 5-ALA is very lower than required dose and, then, over-dose of this photosensitizer should be used to treat cancer effectively [4]. Furthermore, systemic administration of traditional photosensitizers results in their spread throughout the whole body since most of the traditional photosensitizers have no specificity against cancer cells and non-specific accumulation in the whole body [13]. Due to these problems, normal tissues and/or skin frequently exposed to burning and/or pigmentation require that patients avoid sunlight for a long time [13,14,15].

Alternative photosensitizers based on nanotechnology have been developed to solve these drawbacks of traditional photosensitizers [16,17,18,19,20]. Various delivery vehicles including liposomes, proteins, nanomaterials, synthetic polymers, and natural polysaccharide have been suggested to improve PDT efficacy against tumor and minimize unwanted side effects [16,17,18,19,20]. For example, Oliveira reported that photosensitizer-incorporated polymer-liposomes efficiently reduce the viability of T98G glioblastoma multiforme cells [16]. Nanomaterials such as graphene oxide (GO) also significantly improve intracellular uptake of Ce6 and then efficiently inhibited the viability of cancer cells compared to free Ce6 [18]. GO-Ce6 nanocomposites also efficiently targeted tumor tissue rather than normal tissues or organs [18]. Furthermore, nanophotosensitizers using synthetic or natural polymers can be used to improve tumor targeting through ligand specific or stimuli-specific manner [19,20]. Ryu et al. reported that nanophotosensitizers of mPEG-Ce6 conjugates significantly improves aqueous solubility and tumoral uptake of Ce6 in animal tumor xenograft model of colon cancer [21] Furthermore, they argued that nanophotosensitizers efficiently absorbed on the mucosal layer of the porcine colon explants [21]. However, their nanophotosensitizers are not specifically responsive to the physiological peculiarity of tumor microenvironment. Since the tumor microenvironment is quite different from its normal counterpart, nanophotosensitizers should be designed to be sensitive to the tumor microenvironment. For example, abnormalities of microenvironment of tumor tissues such as acid pH and higher ROS levels than those of normal tissues induce higher proliferation rate, migration or metastasis and angiogenesis cancer cells [22,23]. Therefore, nanophotosensitizers should be designed to be sensitive to oxidative stress in tumor tissues [24,25].

In this study, we synthesized mPEGseseCe6PBAP conjugates to fabricate ROS-sensitive nanophotosensitizers and then PDT of HeLa human cervical cancer cells. mPEGseseCe6PBAP conjugates may easily formed nanoscale vehicles since they have amphiphilic structure such as mPEG as a hydrophilic segment and Ce6-PBAP as a hydrophobic segment. Furthermore, diselenide linkage and PBAP is known to be degraded or disintegrated in the presence of ROS, so nanophotosensitizers composed of mPEGseseCe6PBAP conjugates may have ROS-sensitive behavior in an aqueous environment. We studied physicochemical properties and ROS-sensitive behavior of mPEGseseCe6PBAP nanophotosensitizers.

## 2. Materials and Methods

### 2.1. Chemicals

Methoxy poly(ethylene glycol)-succinimidyl succinate (mPEG-NHS) having molecular weight of 5000 g/mol was purchased from SunBio Co., Ltd., (Seoul, Korea). Ce6 was purchased from Frontier Sci. Co. (Logan, UT, USA). 4-(Aminomethyl)phenylboronic acid pinacol ester hydrochloride (PBAP), N-(3-dimethylaminopropyl)-N’-ethylcarbodiimide hydrochloride (EDAC), N-hydroxy succinimide (NHS), triethylamine (TEA), 3-(4,5-dimethyl-2-thiazolyl)-2, 5-diphenyl-2H-tetrazolium bromide (MTT), selenocystamine dihydrochloride, 2’,7’-dichlorofluorescin diacetate (DCFH-DA) and hydrogen peroxide (H_2_O_2_) were purchased from Sigma Aldrich Chem. Co. (St. Louis, MO, USA). Dialysis membranes (Molecular weight cutoffs (MWCO): 2000 Da) were obtained from Spectrum Labs., Inc. (Rancho Dominguez, CA, USA).

### 2.2. Synthesis of mPEGseseCe6 Conjugates

mPEG-sese conjugates: mPEG-NHS (500 mg, 0.1 mM) in 10 mL DMSO and selenocystamine dihydrochloride (160 mg, 0.5 mM) in 10 mL of DMSO/water mixtures (9/1, *v*/*v*) with equal amount of TEA were mixed and then magnetically stirred for 24 h. TEA with equal molar amount of selenocystamine dihydrochloride was added. The reactants were put into a dialysis membrane (MWCO = 2000 g/mol) for dialysis against deionized water. To remove organic solvents, the dialysis procedure was carried out for two days and water was exchanged 3–4 h intervals. The resulting solution was lyophilized for two days to obtain solid. 

mPEG-sese-Ce6 conjugates: Ce6 (30 mg) in 5 mL of DMSO was mixed with EDAC (9.6 mg) and NHS (5.8 mg), and then stirred for 6 h using magnetic stirrer. After that, mPEG-sese conjugates (262 mg) in 10 mL of DMSO were added and then stirred for 24 h. These solutions were put into a dialysis tube having MWCO of 2000 g/mol to remove organic solvents. Following this, these were dialyzed for two days against deionized water. Dark-green solid was obtained by lyophilization for two days. Resultant solid was named as mPEGseseCe6. The yield of mPEGseseCe6 was 94.3%. Yield was calculated based on weight measurement with following equation. Yield of mPEGseseCe6 = [(weight of mPEGseseCe6)/(feeding weight of mPEG-sese conjugates + feeding weight of Ce6)].

mPEGseseCe6PBAP conjugates mPEGseseCe6 (232 mg), EDAC (15.4 mg), and NHS (9.2 mg) were dissolved in dissolved in 20 mL DMSO and then reacted for 6 h by magnetic stirring. After that, PBAP (21.3 mg, 80 µM) with similar molar amount of TEA was added to this solution. This was reacted for 24 h. After that, reactants were adapted to dialysis procedure to remove organic solvents. Dark-green solid was obtained by lyophilization of these solutions. Final solid was named as mPEGseseCe6PBAP. The yield of mPEGseseCe6PBAP was 93.2%. Yield was calculated based on weight measurement with following equation. Yield of mPEGseseCe6PBAP = [(weight of mPEGseseCe6PBAP)/(feeding weight of mPEGseseCe6 conjugates + feeding weight of PBAP)].

### 2.3. ^1^H Nuclear Magnetic Resonance (NMR) Spectra

500 mHz super conducting Fourier transform (FT)-NMR spectrometer (Varian Unity Inova 500 MHz NB High Resolution FT NMR; Varian Inc., Santa Clara, CA, USA) was used to measure ^1^H NMR spectra of conjugates. Chemicals were dissolved in DMSO or D_2_O/DMSO mixtures to measure ^1^H NMR spectra.

### 2.4. Preparation of mPEGseseCe6PBAP Nanophotosensitizers 

mPEGseseCe6PBAP conjugates (20 mg) dissolved in 5 mL DMSO/water mixtures (4/1, *v*/*v*) was dialyzed against water using dialysis membrane (MWCO: 2000 g/mol) for one day. At 2–3 h intervals, deionized water was exchanged for 24 h and, after that, this solution was used to analyze PDT efficacy. Ce6 contents of nanophotosensitizers was measured as follows: mPEGseseCe6PBAP nanophotosensitizers (5 mg) were distributed into 50 mL phosphate buffered saline (PBS, 0.01M, pH 7.4). H_2_O_2_ (final concentration: 100 mM) was added to this solution for disintegration of nanophotosensitizers. This solution was stirred for 48 h and, after that, diluted 20 times with DMSO to measure Ce6 contents in the nanophotosensitizers. Fluorescence spectrophotometer (excitation wavelength: 407, emission wavelength: 664 nm) (RF-5301PC spectrofluorophometer, Kyoto, Japan) was employed to measure Ce6. Free Ce6 dissolved in DMSO was also diluted with PBS (100 mM H_2_O_2_) to compare.

Ce6 contents (wt.%) = (Ce6 weight/total weight of nanophotosensitizers)/100

Ce6 contents in the mPEGseseCe6PBAP were approximately 9.1% (*w*/*w*).

### 2.5. Transmission Electron Microscope (TEM) 

For morphological observation, TEM (H-7600, Hitachi Instruments Ltd., Tokyo, Japan) was used. The nanophotosensitizer solution was dropped onto the carbon film coated grid. Then, this was dried in room temperature. The morphology of nanophotosensitizers was observed at 80 kV.

### 2.6. Fluorescence Spectrophotometer Measurement 

Fluorescence spectra of nanophotosensitizers was measured with fluorescence spectrofluorophotometer (Shimadzu RF-5301PC spectrofluorophometer, Kyoto, Japan). For measurement, Ce6 concentration in nanophotosensitizers was adjusted as 0.1 mg/mL PBS. H_2_O_2_ was added to this solution and then incubated for 4 h at 37 °C. Following this, fluorescence emission spectra was measured between 500 nm and 800 nm with excitation wavelength of 400 nm. Fluorescence images of same solution were observed with Maestro 2 small animal imaging instrument (Cambridge Research and Instrumentation Inc., Hopkinton, MA, USA).

The critical association concentration (CAC) of nanophotosensitizers was evaluated with pyrene using fluorescence spectrophotometer. Pyrene in acetone was poured into a vial and then acetone evaporated (Final concentration of pyrene: 6.0 × 10^−7^ M). Various concentrations of nanophotosensitizers in 10 mL distilled water was added to each vial. To equilibrate pyrene with nanophotosensitizers, vials were incubated at 65 °C for 3 h and then left to cool 2 h at room temperature. The measurement condition was as follows: Emission wavelength, 390 nm for excitation spectra (excitation and emission bandwiths were 1.5 nm and 1.5 nm, respectively).

### 2.7. Ce6 Release from Nanophotosensitizer

Aqueous nanophotosensitizer solution was adjusted to 1 mg/mL with PBS. 5 mL this solution in dialysis membrane (MWCO = 2000 g/mol) was put into a conical tube with 45 mL of PBS. H_2_O_2_ was added to this solution to study disintegration of nanophotosensitizers and then incubated in a shaker incubator (SI-600R, Jeiotech Co., Daejeon, Korea) at 37 °C and 100 rpm. For measurement of released Ce6, whole media was taken and replaced with fresh media. This solution was used to measure Ce6 concentration with fluorescence spectrofluorophotometer (RF-5301PC spectrofluorophotometer, Kyoto, Japan). The excitation and emission wavelength were 407 nm and 664 nm, respectively. All results were triplicated and expressed as mean ± standard deviation (S.D.).

### 2.8. Cell Culture

Cells used in this study was obtained from Korean Cell Line Bank (Seoul, Korea). HeLa human cervical cancer cells were cultured using MEM medium (Gibco, Grand Island, NY, USA), supplemented with 10% heat-inactivated fetal bovine serum (FBS) (Invitrogen) and 1% penicillin/streptomycin at 37 °C in a 5% CO_2_ incubator. RAW264.7 mouse macrophage cells were cultured in DMEM (Gibco, Grand Island, NY, USA) medium supplemented with 10% FBS (Invitrogen) and 1% penicillin/streptomycin. 

### 2.9. PDT Treatment of Cancer Cells 

PDT treatment was performed as follows: 2 × 10^4^ HeLa cells were seeded in 96 well plates. This was incubated in 5% CO_2_ at 37^o^C. For Ce6 treatment, Ce6 in DMSO was diluted with serum-free media. Final DMSO concentration was adjusted to less than 1% (*v*/*v*)). Nanophotosensitizer solution was sterilized with 0.8 µm syringe filter. Then, this was also diluted with serum-free media. Cells in 96 wells were treated with these solutions for 2 h. Following this, cells were washed with PBS and then replaced with 100 µL phenol red-free media. Expanded homogenous beam (SH Systems, Gwangju, Korea) having 664 nm was used to irradiate cells (2.0 J/cm^2^). Photo-radiometer (DeltaOhm, Padova, Italy) was employed to measure light intensity and dose. The irradiated cells were incubated for 1 day at 5% CO_2_ and 37 °C. Then, MTT proliferation assay was used to measure cell viability. 30 µL MTT solution (5 mg/mL in PBS) was added to 96 wells and further cultured for 4 h. Following this, crystallized formazan in viable cells was dissolved with DMSO (100 µL) and then absorbance at 570 nm was measured with an Infinite M200 pro microplate reader. All procedures were done in dark condition.

Dark toxicity was performed as follows: dark toxicity has been performed with similar procedure as described in PDT without light irradiation.

### 2.10. Intracellular Uptake of Nanophotosensitizers

2 × 10^4^ HeLa cells in 96 wells were cultured overnight in 5% CO_2_ incubator at 37 °C. Ce6 or nanophotosensitizers were exposed to cells for 2 h as similarly described in PDT treatment. After washing of cells with PBS, cells were solubilized in 50 µL of lysis buffer (GenDEPOT, Barker, TX, USA) to measure Ce6 uptake ratio. Infinite M200 pro microplate reader (Tecan) (excitation wavelength: 407 nm, emission wavelength: 664 nm) was employed to measure Ce6 contents in cells.

For observation of cells using fluorescence microscope, HeLa cells (3 × 10^5^) in six wells with cover glass were treated with Ce6 or nanophotosenstitizers. 60 min later, cells were washed with PBS and then fixed with 4% paraformaldehyde for 15 min. This was washed with PBS again to remove byproducts. Mounting solution (Immunomount, Thermo Electron Co., Pittsburgh, PA, USA) was used to immobilize these cells. After that, fluorescence microscope (Eclipes 80i; Nikon, Tokyo, Japan) was used to observe cells.

### 2.11. Flow Cytometry

Ce6 or nanophotosensitizers (2 μg/mL Ce6 concentration) treated to HeLa cells (1 × 10^6^) in 6 well plates for 60 min and then they were washed with PBS twice. Cells were harvested by trypsinization and then Invitrogen Attune NxT flow cytometers (ThermoFisher Scientific, Waltham, MA, USA) was employed to measure cells.

### 2.12. ROS Generation Assay 

HeLa cells (2 × 10^4^) in 96-wells were cultured overnight in 5% CO_2_ incubator. Ce6 or nanophotosensitizers in the phenol red free media treated to cells as similarly as described in PDT treatment. After that, DCFH-DA in PBS (final concentration: 20 µM) was added to cell culture and then incubated for 2 h at 37 °C. After washing of cells with PBS, 100 µL fresh phenol red free media was added to irradiate cells at 664 nm (2.0 J/cm^2^). Infinite M200 pro microplate reader (Tecan Trading AG, Männedorf, Switzerland) was employed to measure intracellular ROS level (excitation wavelength, 485 nm; emission wavelength, 535 nm).

### 2.13. In Vivo Animal Imaging

Five weeks old nude BALb/C mice (20 g) was used to observe fluorescence images of tumor. HeLa cells (1 × 10^6^) were subcutaneously (s.c.) injected to the back of mice when diameter of tumor mass became larger than 6 mm. Nanophotosensitizer solution was intravenously (i.v.) injected to the tail vein (Injection volume, 100 µL; dose, 10 mg/kg as a Ce6 concentration) of mice. Expanded homogenous beam (SH Systems, Gwangju, Korea) was employed to irradiate mice. On the 3 h and 6 h after the drug injection, one mouse was exposed to light irradiation at 664 nm with 5.0 J/cm^2^. Another one was also placed on the field of light irradiation with shielding of tumor mass to avoid light irradiation. 9 h after the drug injection, MaestroTM 2 small animal imaging instrument (Cambridge Research and Instruments, Inc. Woburn, MA, USA) was employed to observe biodistribution of nanophotosensitizers.

### 2.14. Statistical Analysis 

The statistical significance of the results was analyzed with Student’s test using SigmaPlot^®^ program.

## 3. Results

### 3.1. Synthesis of mPEGseseCe6PBAP Conjugates

**Figure 1:**
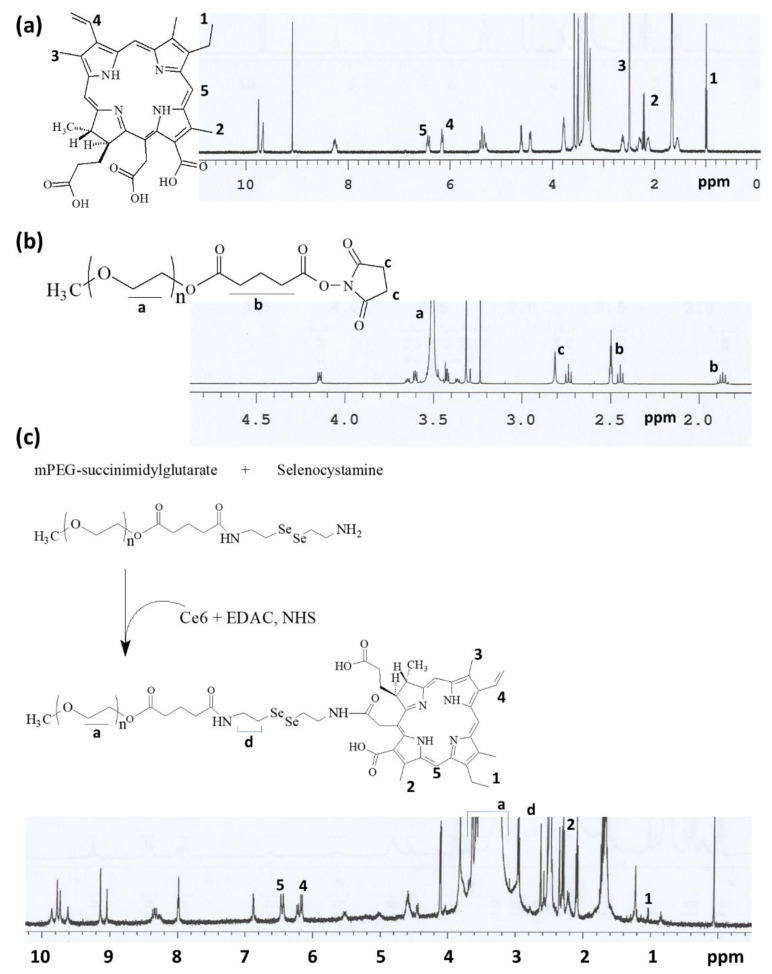
Chemical structure and ^1^H NMR spectra of (**a**) Ce6; (**b**) mPEG-NHS. (**c**) Synthesis scheme and ^1^H NMR spectra of mPEGseseCe6 conjugates.

**Figure 2:**
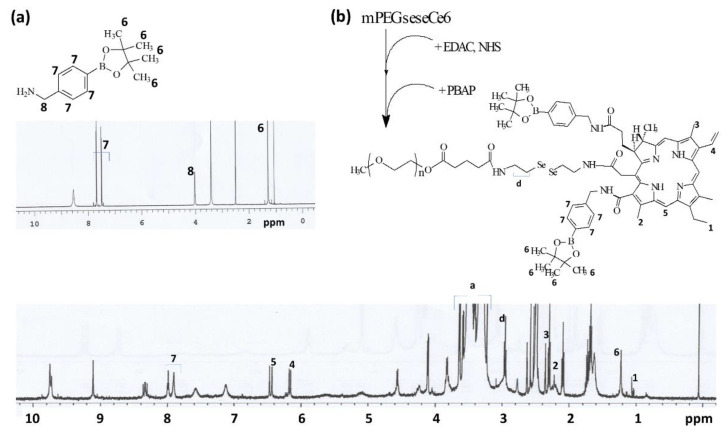
Chemical structure, synthesis scheme and ^1^H NMR spectra of PBAP (**a**) and mPEGseseCe6PBAP conjugates (**b**).

The synthesis scheme of mPEGseseCe6PBAP conjugates was shown in Figure 1. Specific peaks of Ce6 were confirmed between 1.0~10 ppm (Figure 1a) while mPEG succinimidyl glutarate showed specific peaks at 3.5–3.6 ppm of ethylene group and 1.6~2.8 ppm of succinimidyl glutarate group (Figure 1b). As shown in Figure 1c, mPEG succinimidyl glutarate was conjugated with selenocystamine to endow diselenide group and amine end group. Following this, Ce6 was attached to amine end group of mPEGsese conjugates. Remained two carboxylic group of Ce6 were activated with EDAC/NHS. Then, this was conjugated with PBAP (Figure 2). PBAP peaks were confirmed at 1.2–1.4 ppm, 4.0 ppm and 7.4–7.8 ppm (Figure 2a). All of specific peaks of mPEGseseCe6PBAP was confirmed (Figure 2b); peaks of conjugates (mPEG, selenocystamine, Ce6 and PBAP) were confirmed between 1.0~8.0 ppm. Yield of final product (mPEGseseCe6PBAP conjugates) was estimated by weight measurement as 93.2%. From the weight measurement, more than 1.8 PBAP was attached to mPEGseseCe6.

### 3.2. Fabrication and Characterization of mPEGseseCe6PBAP Nanophotosensitizers

Nanophotosensitizers of mPEGseseCe6PBAP conjugates were fabricated by dialysis procedure. As shown in Table 1, Ce6 contents in mPEG-sese-Ce6 conjugates and nanophotosensitizers of mPEGseseCe6PBAP conjugates were 10.1 and 9.1% (*w*/*w*), respectively. The experimental Ce6 contents was slightly lower than theoretical value. Conjugation of Ce6 into mPEGseseCe6PBAP conjugates has done properly even though its experimental value was slightly lower than theoretical value.

**Figure 3:**
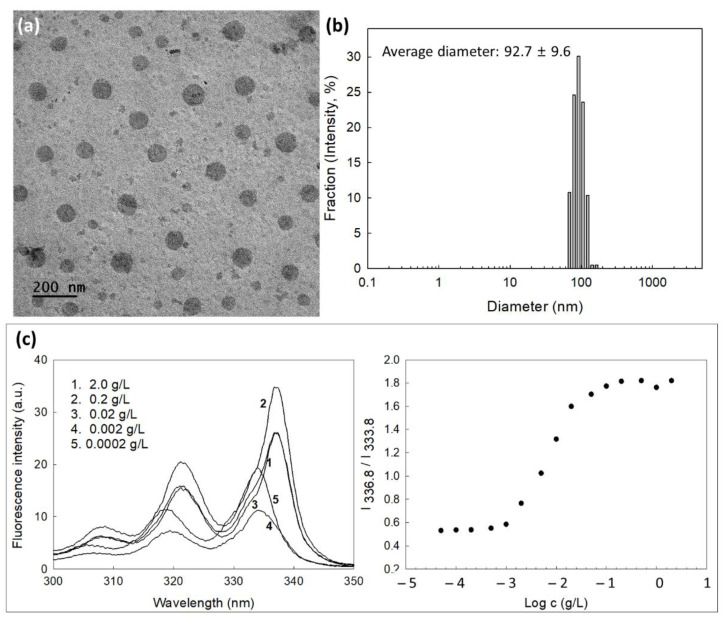
(**a**) Morphological observation of nanophotosensitizers of mPEGseseCe6PBAP conjugates by TEM. (**b**) Typical particle size distribution of nanophotosensitizers of mPEGseseCe6PBAP conjugates. Average particle sizes were mean±standard deviation from five independent analysis. (**c**) Fluorescence excitation spectra of pyrene/mPEGseseCe6PBAP nanophotosensitizers in distilled water (Emission wavelength: 390.0 nm) (**left**) and I_336.8_/I_333.8_ intensity ratio plots from pyrene excitation spectra vs. log *c* for mPEGseseCe6PBAP nanophotosensitizers in distilled water (**right**).

Since mPEG is a hydrophilic polymer and Ce6 or PBAP is a hydrophobic chemical, self-assembled nano-vehicles can be formed in the aqueous solution by mPEGseseCe6PBAP conjugates. They have spherical shapes with small diameter less than 100 nm (Figure 3a). Their average particle sizes were 92.7 ± 9.6 nm and they have narrow size distributions (Figure 3b), indicating that spherical nanoparticles formed by mPEGseseCe6PBAP conjugates in aqueous solution. Since mPEGseseCe6PBAP conjugates have amphiphilic properties, they can be assembled in nanosized particles in aqueous solution in critical concentration. Therefore, critical association concentration (CAC) was evaluated in aqueous solution as shown in Figure 3b. Fluorescence excitation spectra of pyrene (6.0 × 10^−7^ M) was measured in the presence mPEGseseCe6PBAP nanophotosensitizers. When concentration of nanophotosensitizers was increased, a red shift of pyrene was observed as shown in Figure 3c (left). Then, the (0,0) bands in the pyrene excitation spectra were compared in the intensity ratio I_336.8_/I_333.8_ as shown in Figure 3c (right). Cross-over region between flat region at low concentration of nanophotosensitizers and sigmoidal region was noted as a CAC value of nanophotosensitizers. The CAC value of mPEGseseCe6PBAP nanophotosensitizers was approximately 0.003 g/L.

**Figure 4:**
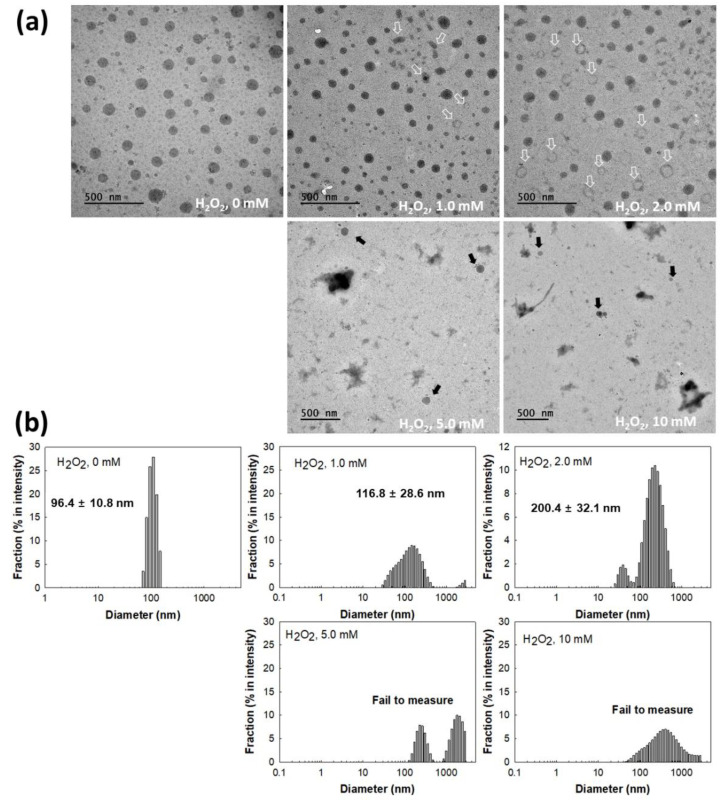
(**a**) H_2_O_2_ effect on the changes of nanophotosensitizer morphologies. (**b**) H_2_O_2_ effect on the size distribution changes of nanophotosensitizers.

Figure 4 shows the changes of mPEGseseCe6PBAP nanophotosensitizers by oxidative stress. To study oxidative stress, H_2_O_2_, a typical ROS, added to nanophotosensitizer solution. As shown in Figure 4a, morphology of nanophotosensitizers were changed from spherical shape to irregular form or hollow shape according to H_2_O_2_ concentration (open arrows in Figure 4a. Especially, morphology of nanophotosensitizers were significantly disintegrated at 10 mM H_2_O_2_ even though some nanophotosensitizers still maintained their spherical shapes. Furthermore, the particle size changes of nanophotosensitizers also showed similar results in the presence of H_2_O_2_. Figure 4b shows that size distribution of nanophotosensitizers became broad and multimodal patterns at 1.0 mM or 2.0 mM H_2_O_2_. However, particle size measurement was practically failed at higher H_2_O_2_ concentrations (Figure 4b), indicating that mPEGseseCe6PBAP nanophotosensitizers have sensitivity against oxidative stress in biological environment.

**Figure 5:**
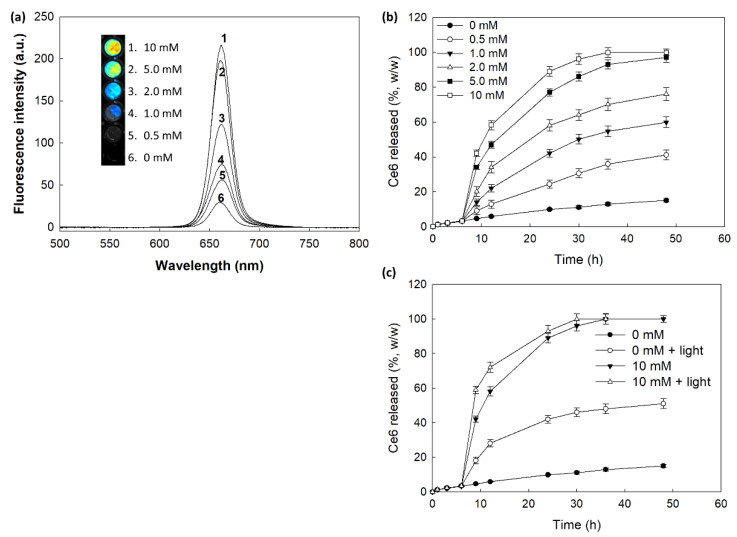
(**a**) The changes of fluorescence intensity according to the concentration of H_2_O_2_. For fluorescence spectra, nanophotosensitizers were incubated for 4 h at 37 °C with or without H_2_O_2_. Ce6 concentration in nanophotosensitizers: 0.1 mg/mL PBS. (**b**) Ce6 release from nanophotosensitizers. To investigate oxidative stress on the Ce6 release, H_2_O_2_ was added to media at 6 h of release experiment. (**c**) The effect of light irradiation on the Ce6 release from nanophotosensitizers. H_2_O_2_ was added to release media at 6 h of release experiment and then nanophotosensitizers was exposed to light irradiation at 664 nm with 5.0 J/cm^2^ using an expanded homogenous beam.

Figure 5 shows the changes of fluorescence intensity and Ce6 release behavior. As shown in Figure 5a, H_2_O_2_ in the aqueous solution of nanophotosensitizers significantly increases fluorescence intensity (i.e., higher concentration of H_2_O_2_ induced increase of fluorescence intensity). Furthermore, Ce6 release rate from nanophotosensitizers became higher by addition of H_2_O_2_ in the aqueous solution than those in the absence of H_2_O_2_. Furthermore, Ce6 release from nanophotosensitizers also increased when nanophotosensitizers were irradiated by light as shown in Figure 5c. These results indicated that mPEGseseCe6PBAP nanophotosensitizers have light and ROS-sensitivity. Then, Ce6 release was increased with light and ROS-sensitive manner.

### 3.3. PDT Study Using Cell Culture

**Figure 6:**
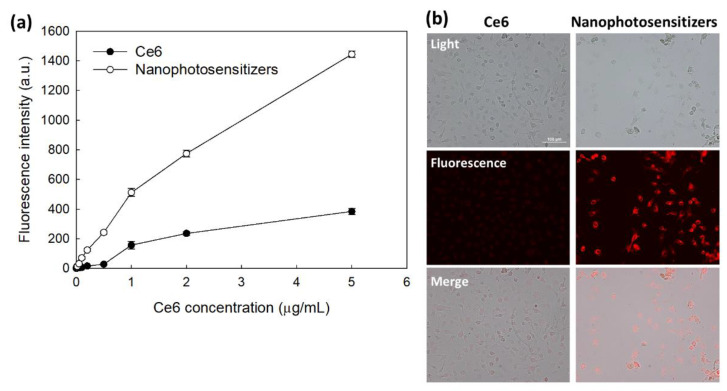
(**a**) Ce6 uptake ratio of HeLa cells. 2 × 10^4^ HeLa cells seeded in 96 well plates were exposed to Ce6 or nanophotosensitizersas for 2 h. (**b**) Fluorescence microscope images of HeLa cells treated with Ce6 or mPEGseseCe6PBAP nanophotosensitizers (Bar = 100 µm). For fluorescence microscopic observation, 3 × 10^5^ HeLa cells seeded in six well plates with cover glass were exposed Ce6 or nanophotosenstitizer for 60 min. Magnification: × 200.

The Ce6 uptake ratio by HeLa cells was shown in Figure 6. Figure 6a shows that increase of Ce6 concentration resulted in Ce6 uptake both free Ce6 and nanophotosensitizers. Especially, significantly higher Ce6 uptake ratio was observed by treatment of nanophotosensitizers compared to free Ce6 (Figure 6a). Ce6 uptake ratio of nanophotosensitizers was almost three times higher than that of free Ce6. Morphological observation of cells using fluorescence microscope also showed similar results (i.e., red fluorescence intensity was significantly higher by treatment of nanophotosensitizer than that of free Ce6 treatment (Figure 6b)). These results indicated that nanophotosensitizers were efficiently internalized intracellularly rather than free Ce6. Furthermore, H_2_O_2_ was added to cell culture to evaluate ROS-sensitivity of nanophotosensitizers. 

**Figure 7:**
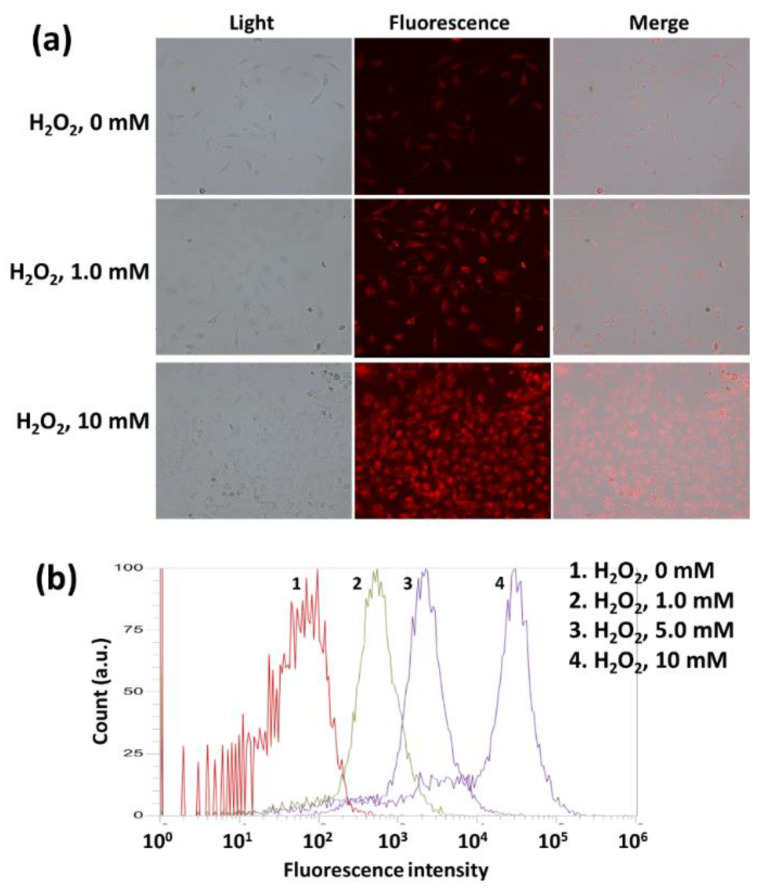
The effect of H_2_O_2_ addition on the nanophotosensitizer delivery in HeLa cells. (**a**) Fluorescence microscope images. (**b**) Flow cytometry. Ce6 or nanophotosensitizers (2 μg/mL Ce6 concentration) treated to HeLa cells (1 × 10^6^) in six well plates for 60 min with or without H_2_O_2_. Magnification: × 200.

The effect of H_2_O_2_ on the Ce6 uptake of HeLa cells was shown in Figure 7. The higher the H_2_O_2_ concentration induced the higher the intracellular fluorescence intensity (Figure 7a). Furthermore, flow cytometric analysis showed that H_2_O_2_ addition in cell culture resulted in increase of fluorescence intensity (Figure 7b). These results indicated that oxidative stress in biological system accelerates intracellular delivery of mPEGseseCe6PBAP nanophotosensitizers through ROS-sensitive delivery manner. Furthermore, these results also indicated that intracellular delivery and disintegration of mPEGseseCe6PBAP nanophotosensitizers can be controlled by oxidative stress in the biological system.

**Figure 8:**
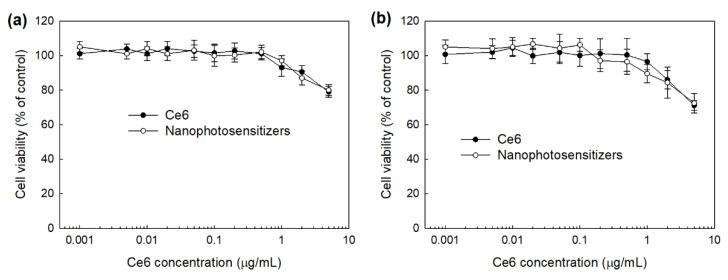
Intrinsic cytotoxicity of free Ce6 or mPEGseseCe6PBAP nanophotosensitizers against RAW264.7 cells (**a**) and HeLa cells (**b**). Free Ce6 or mPEGseseCe6PBAP nanophotosensitizers treated to RAW264.7 mouse macrophage cells or HeLa human cervical cancer cells in the absence of light.

The intrinsic toxicity of mPEGseseCe6PBAP nanophotosensitizers were evaluated with RAW264.7 mouse macrophage cells and HeLa human cervical cancer cells (Figure 8). The viability of RAW264.7 cells was higher than 80% until 2 µg/mL and 79% at 5 µg/mL Ce6 concentration (Figure 8a). Nanophotosensitizers showed almost similar cytotoxicity compared to Ce6 (Figure 8a); cell viability was higher than 80% until 5 µg/mL Ce6 concentration. HeLa cell viability was also higher than 80% until 2 µg/mL Ce6 concentration both free Ce6 and mPEGseseCe6PBAP nanophotosensitizers (Figure 8b), indicating that nanophotosensitizers have low intrinsic cytotoxicity in the absence of light irradiation as well as free Ce6. As well as free Ce6, mPEGseseCe6PBAP nanophotosensitizers have no toxicity until 2 µg/mL Ce6 concentration.

**Figure 9:**
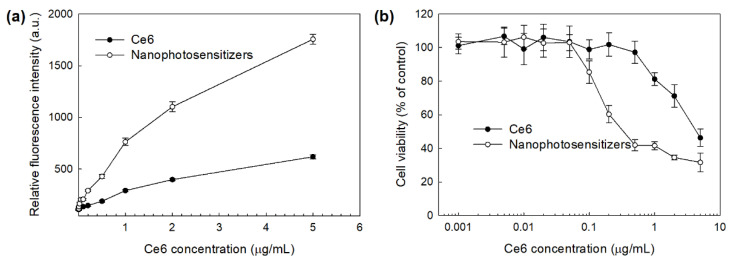
PDT efficacy of Ce6 and mPEGseseCe6PBAP nanophotosensitizers against HeLa human cervical cancer cells. (**a**) intracellular ROS generation; (**b**) PDT against HeLa cells. DCFH-DA assay was employed to measure ROS generation in cells. Cells (2 × 10^4^ cells/well) were irradiated at 664 nm (2 J/cm^2^).

Figure 9 shows the PDT efficacy of nanophotosensitizers. The intracellular ROS generation was dose-dependently increased both of free Ce6 and mPEGseseCe6PBAP nanophotosensitizers (Figure 9a). Especially, ROS generation in the treatment of nanophotosensitizers was three times higher than that of free Ce6. Figure 9b shows the PDT efficacy of Ce6 and mPEGseseCe6PBAP nanophotosensitizers against HeLa cells. Cell viability under light irradiation was dose-dependently decreased at higher than 0.2 µg/mL concentration of Ce6 treatment and 0.05 µg/mL concentration of nanophotosensitizers (Figure 9b. Especially, treatment of mPEGseseCe6PBAP nanophotosensitizers efficiently inhibited cell viability rather than free Ce6 treatment, indicating that higher efficacy in intracellular delivery, ROS generation, and PDT can be obtained by the use of nanophotosensitizers.

### 3.4. Animal Tumor Imaging Using Tumor Xenograft Model

**Figure 10:**
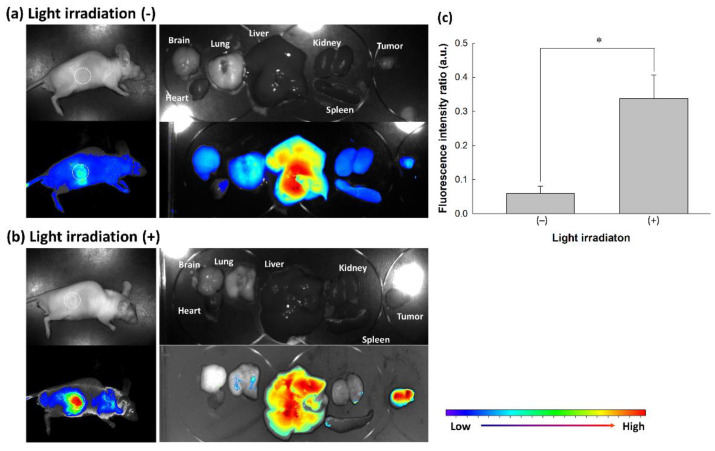
Animal tumor imaging of tumor xenograft model. Tumor xenograft model of HeLa cells were prepared in the back of nude BALb/C mice. Nanophotosensitizer solution was i.v. administered through tail vein (Injection volume: 100 µL; dose, 10 mg/kg as a Ce6 concentration). On the 3 h and 6 h after the drug injection, one mouse was exposed to light irradiation at 664 nm with 5.0 J/cm^2^ using an expanded homogenous beam. Another one was also placed on the field of light irradiation with shielding of tumor mass to avoid light irradiation. Nine hours after the drug injection, mice were sacrificed and observed. Animal tumor xenograft (**a**) without light irradiation and (**b**) with light irradiation. (**c**) Comparison of fluorescence intensity between tumor and liver. Fluorescence intensity between tumor and liver was calculated from three mouse and expressed as mean ± S.D. Fluorescence intensity ratio (arbitrary units) = fluorescence intensity of tumor/fluorescence intensity of liver. * *p* < 0.01.

To evaluate the effect of oxidative stress using in vivo tumor xenograft model, nanophotosensitizers were i.v. administered into the tail vein of mice. Figure 10 shows that fluorescence intensity in the liver was significantly stronger than that of other organs. Especially, fluorescence intensity in the tumor mass was also stronger in the light irradiation (Light irradiation (+), Figure 10b) than that in the absence of light irradiation (Light irradiation (−), Figure 10a). As shown in Figure 10c, light irradiation induced increase of fluorescence intensity in tumor tissue when fluorescence intensity between the liver and tumor was compared (i.e., relative fluorescence intensity of tumor tissue vs. liver was significantly higher by light irradiation (Light irradiation (+)) than that in the absence of light irradiation (Light irradiation (−))). These results indicated that mPEGseseCe6PBAP nanophotosensitizers have ROS-sensitivity in vitro cell culture model and in vivo of tumor model. Then, they can be delivered, disintegrated, and photoactivated under light-irradiated oxidative stress. 

## 4. Discussion

Cervical cancer, a third most common cancer in women, is derived from cervix [12,26,27,28]. Surgical removal, chemotherapy, and/or radiotherapy are the most common treatment modalities for cervical cancer [29,30]. The recurrence rate after radical hysterectomy, which is believed to be a curative option, is higher than 10% [31,32]. To manage primary and/or recurrent cervical cancer, adjuvant chemotherapy has been explored [33,34,35,36]. Chemotherapeutic agents such as paclitaxel, ifosfamide, and cisplatin give advantages in prolongation of median survival times [33]. However, systemic chemotherapy for various types of cancers is always faced with unwanted systemic toxicity against normal cells or tissues (i.e., side effects such as tumor resistance, nephrotoxicity, neurotoxicity, hematological toxicity, and bone marrow depression are always problematic in clinical approaches chemotherapeutic agents) [34,35]. From these points of view, PDT can be considered as a promising option for cervical cancer since photosensitizers for PDT of cancer have negligible cytotoxicity against normal cells in the absence of light irradiation [36]. Especially, photosensitizers produce excessive ROS in the field of light irradiation and then they can be applicable to target or local therapy of tumor. Therefore, these intrinsic properties of PDT regimen enable one to minimize systemic cytotoxicity for patients. However, PDT using small molecular weight photosensitizers has limitations in the penetration depth of light irradiation; irradiated light to human tissues is unable to approach deeper than 15 mm [5,6]. Otherwise, these properties of PDT regimen are considered as a promising treatment option for cervical cancer. Particularly, squamous cell carcinoma (SCC) is the main composition of cervical cancers in morphological classification (SCC is about 90% and mis-adenocarcinoma is about 10%) [28,29]. For example, PDT of early stages of cervical cancer using Photogem^®^ induced significant decrease in pre-cancerous lesions [37]. Also, the PDT approach using Porfimer sodium against cervical cancer induced complete regression in 96.4% of patients [38]. In our results, both normal cells and cancer cells was not significantly affected by treatment of free Ce6 or nanophotosensitizers without light irradiation while the presence of light irradiation resulted in dose-dependent death of cancer cells. Particularly, nanophotosensitizers have improved PDT efficacy against cervical cancer cells. These effects were attained by improved uptake by cancer cells and enhanced ROS production. As shown in Figure 6, nanophotosensitizers showed higher Ce6 uptake ratio compared to free Ce6. Since free Ce6 as a small molecule has anionic charge in the physiological environment, it seems to be that Ce6 has relatively lower uptake efficacy than nanophotosensitizers. For example, Soukos et al., reported that Ce6 and poly-L-lysine conjugates having polycationic, polyanionic and neutral conjugates showed different Ce6 uptake efficiency [39]. They argued that Ce6 uptake ratio of polymer conjugates having neutral charges was higher than free Ce6. In our results, uptake ratio of nanophotosensitizers was three-times higher than that of free Ce6. These results indicated that nanophotosensitizers have higher potential in intracellular delivery of photosensitizers than that of small molecules.

Nano-dimensional vehicles have been spotlighted in the biomedical field for the last few decades due to their potential in site-specific drug delivery and drug targeting since they have advantages such as small particle size, incorporation/solubilization of hydrophobic drug, and ease of administration [40,41]. Due to these peculiarities, nanoparticles can be synthesized or fabricated to be sensitive to abnormal physiological status in human body [18,19]. For example, Duan et al., reported that nanomedicine based on glycopolymer-pyropheophorbide a conjugates and poly[*N*-(2-hydroxypropyl) methacrylamide have superior specificity against tumor cells [42]. Furthermore, their group also reported that supramolecular self-assemblies of dendritic peptides possess high stability to prolong blood circulation and enhance tumor retention compared to pyropheophorbide a [43].

Otherwise, the abnormal physiological state of the tumor microenvironment is distinguished from normal counterpart (i.e., the tumor is characterized by overexpression of various receptors, acidic pH, increased metabolism, and higher reduction/oxidation (redox) potential rather than normal cells or tissues) [22,23,24,25]. Then, abnormalities of the tumor microenvironment endow nanoparticles to be sensitive to tumor. Particularly, nanoparticles having ROS-sensitivity gives an opportunity to target tumor tissue to maximize anticancer drug exposure in the site of higher ROS level [44,45]. Jang et al. reported that biodegradable nanofiber mats having diselenide linkages have responsiveness against ROS and then specifically release anticancer drug according to ROS level in aqueous solution [45]. In our study, nanophotosensitizers also responded to oxidative stress and then were disintegrated by oxidative stress (Figure 4). Furthermore, oxidative stress in aqueous environment accelerates release rate of photosensitizers from nanophotosensitizers (Figure 5). Particularly, light irradiation may accelerate disintegration of mPEGseseCe6PBAP nanophotosensitizers and Ce6 release rate. Actually, H_2_O_2_ addition to cell culture increased intracellular Ce6 uptake ratio by increased oxidative stress as shown in Figure 7. This result also supported by in vivo animal tumor imaging study (i.e., tumor tissues with light irradiation showed higher fluorescence intensity than other organs while the absence of light irradiation did not increase fluorescence intensity in tumor tissue; Figure 10). These results indicated that nanophotosensitizers of mPEGseseCe6PBAP conjugates were ROS-sensitive nano-carriers for photosensizers and then their delivery capacity can be improved by oxidative stress in tumor tissues.

## 5. Conclusions

Nanophotosensitizers of mPEGseseCe6PBAP conjugates were synthesized for ROS-sensitive delivery of Ce6 to tumors. Nanophotosensitizers of mPEGseseCe6PBAP conjugates were prepared by the dialysis method. Nanophotosensitizers revealed spherical shapes with 92.7 ± 9.6 nm in average particle size. Nanophotosensitizers were sensitively disintegrated against H_2_O_2_ concentration and their monomodal pattern in size distribution was changed to a multimodal pattern. Furthermore, Ce6 release from nanophotosensitizers also increased according to the H_2_O_2_ concentration. Compared to free Ce6, nanophotosensitizers showed improved Ce6 uptake ratio in HeLa cells in vitro. In the presence of H_2_O_2_, intracellular Ce6 uptake of nanophotosensitizers were sensitively increased according to H_2_O_2_ concentration. These resulted indicated the ROS-sensitivity of mPEGseseCe6PBAP nanophotosensitizers and their delivery capacity can be improved against cancer cells. As well as free Ce6, the absence of light irradiation induced low cytotoxicity against RAW264.7 cells and HeLa cells in the treatment of nanophotosensitizers. However, light irradiation induced cell death with dose-dependent manner. Especially, nanophotosensitizers showed significantly higher ROS generation and phototoxicity against HeLa cells in vitro. In vivo animal imaging study after intravenous administration of nanophotosensitizers showed that light irradiation induced higher fluorescence intensity in tumor tissue than that of other tissues. Otherwise, the absence of light irradiation did not increase fluorescence intensity in tumor tissues. These results proved that nanophotosensitizers respond to oxidative stress of tumor tissue, release Ce6 from nanophotosensitizers with ROS-sensitivity, reveal improved cell death under light irradiation and target tumor tissue under light irradiation. We suggest that mPEGseseCe6PBAP nanophotosensitizers are promising candidates for PDT of cervical cancer.

## Figures and Tables

**Table 1:** Particle size and Ce6 contents in the nanophotosensitizers.

	Drug Contents (%, *w*/*w*)		Particle Size (nm)
	Theoretical ^a^	Experimental ^b^	
mPEG-sese-Ce6 conjugates Nanophotosensitizers	10.3 9.6	10.1 9.1	- 92.7 ± 9.6

^a^ Theoretical content was calculated from molecular weight. ^b^ Experimental content was measured as depicted in Materials and methods section.

## Data Availability

The data presented in this study are contained within the article.
